# Determination of ceftriaxone in human plasma using liquid chromatography–tandem mass spectrometry

**DOI:** 10.12688/wellcomeopenres.15141.2

**Published:** 2021-09-03

**Authors:** Thamrong Wongchang, Markus Winterberg, Joel Tarning, Natthida Sriboonvorakul, Sant Muangnoicharoen, Daniel Blessborn

**Affiliations:** 1 Mahidol-Oxford Tropical Medicine Research Unit (MORU), Faculty of Tropical Medicine, Mahidol University, Bangkok, 10400, Thailand; 2 Department of Clinical Tropical Medicine, Faculty of Tropical Medicine, Mahidol University, Bangkok, 10400, Thailand; 3 Centre for Tropical Medicine & Global Health, Nuffield Department of Medicine, University of Oxford, Oxford, OX3 7FZ, UK

**Keywords:** Ceftriaxone, bioanalytical method, human plasma, liquid chromatography tandem mass spectrometry

## Abstract

Ceftriaxone is a cephalosporin antibiotic drug used as first-line treatment for a number of bacterial diseases. Ceftriaxone belongs to the third generation of antibiotics and is available as an intramuscular or intravenous injection. Previously published pharmacokinetic studies have used high-performance liquid chromatography coupled with ultraviolet detection (HPLC-UV) for the quantification of ceftriaxone. This study aimed to develop and validate a bioanalytical method for the quantification of ceftriaxone in human plasma using liquid chromatography followed by tandem mass spectrometry (LC-MS/MS). Sample preparation was performed by protein precipitation of 100 µl plasma sample in combination with phospholipid-removal techniques to minimize matrix interferences. The chromatographic separation was performed on an Agilent Zorbax Eclipse Plus C18 column with 10 mM ammonium formate containing 2% formic acid: acetonitrile as mobile phase at a flow rate of 0.4 ml/min with a total run time of 10 minutes. Both the analyte and cefotaxime (internal standard) were quantified using the positive electrospray ionization (ESI) mode and selected reaction monitoring (SRM) for the precursor-product ion transitions *m/z* 555.0→396.1 for ceftriaxone and 456.0→324.0 for cefotaxime. The method was validated over the concentration range of 1.01-200 μg/ml. Calibration response showed good linearity (correlation coefficient > 0.99) and matrix effects were within the ±15% limit in 6 different lots of sodium heparin plasma tested. However, citrate phosphate dextrose plasma resulted in a clear matrix enhancement of 24% at the low concentration level, which was not compensated for by the internal standard. Different anticoagulants (EDTA, heparin and citrate phosphate dextrose) also showed differences in recovery. Thus, it is important to use the same anticoagulant in calibration curves and clinical samples for analysis. The intra-assay and inter-assay precision were less than 5% and 10%, respectively, and therefore well within standard regulatory acceptance criterion of ±15%.

## Introduction

Antibiotic resistance development is a serious global health concern. The number of deaths from drug-resistant infections is predicted to increase from 700,000 to 10 million deaths annually by 2050 with an estimated cost of up to US$ 100 trillion ^1, 2^. The impact of resistance will increase patient mortality, morbidity, length of hospitalization, and health-care costs ^3, 4^. Furthermore, development of widespread antibiotics resistance decreases the number of effective antibiotics rapidly, and new drug discovery of novel drugs are not delivering new agents in sufficient rate to combat this rapidly increasing issue ^5^. Therefore, all strategies to preserve efficacy of available drugs should be considered. Only with an in-depth understanding of the pharmacokinetic and pharmacodynamic (PK/PD) properties of a drug, can we achieve an evidence-based dosing (i.e. right drug, at the right dose and time). However, accurate and reliable bioanalytical methods for drug determination is a fundamental element to obtain reliable pharmacokinetic data.

Ceftriaxone is an important antibiotic drug that has been used as a first-line treatment for a number of bacterial infectious diseases for more than 30 years. Although the drug was discovered in the 1980s by Hoffmann-La Roche, some PK/PD properties, particularly in neonates, have not been well defined. Published pharmacokinetic studies were mostly performed in adults, excluding populations such as neonates with severe infections, infants, and malnourished young children ^6– 10^. To be able to perform PK/PD studies on these groups, a sensitive and selective bioanalytical method is needed.

Most of the previously published methods for ceftriaxone determination were performed by high performance liquid chromatography coupled with ultraviolet detection (HPLC-UV) ^6– 8, 11, 12^, which is less sensitive and requires larger sample volume compared to LC-MS/MS assays. The large sample volumes required for the HPLC-UV detection render these assays inappropriate for measuring drug levels in neonates, infants and young children. Another drawback of the HPLC-UV techniques are long analysis times, often 10 to 20 minutes per sample.

The objective of this study was to develop and validate an accurate and sensitive bioanalytical method for ceftriaxone determination in low volume human plasma using LC-MS/MS. Only a few research publications have reported using LC-MS/MS for ceftriaxone determination in human biological samples ^13– 16^. Thus, this will be one of the first methods for ceftriaxone determination by LC-MS/MS and an alternative option to the already published methods.

## Methods

### Materials and reagents

**Figure 1.**
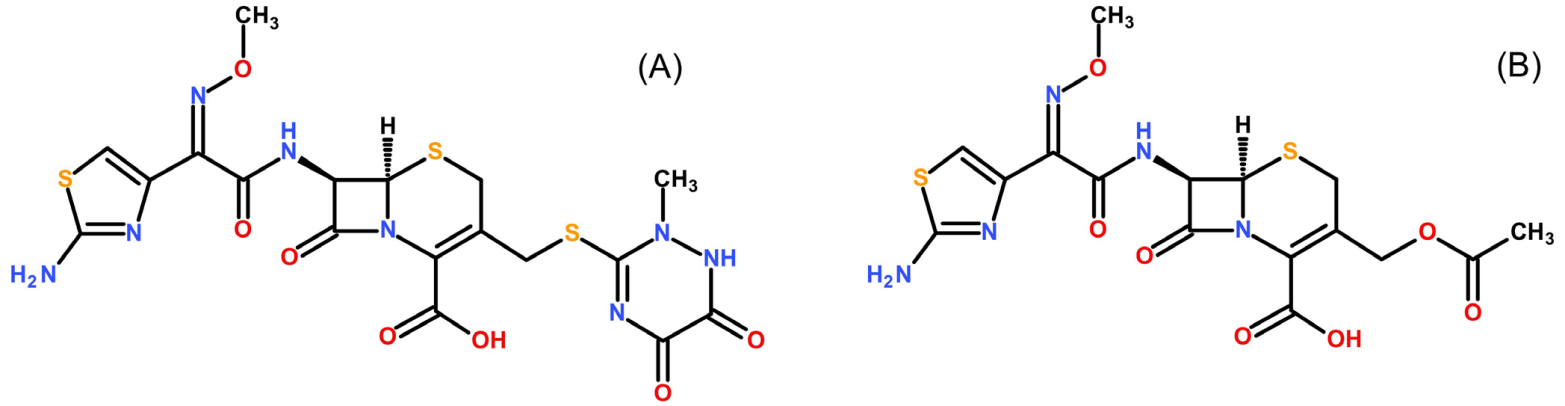
Molecular structures. Structures of ceftriaxone ( **A**) and the internal standard cefotaxime ( **B**) are shown.

Ceftriaxone disodium salt was supplied by Sigma-Aldrich Chemicals (St Louis, MO, USA). The internal standard, cefotaxime sodium salt, was from Santa Cruz Biotechnology (Dallas, TX, USA). Ceftriaxone-D _3_ disodium salt hydrate was supplied by Medical Isotopes, Inc. (Pelham, NH, USA). Figure 1 shows the molecular structures of ceftriaxone and cefotaxime. Formic acid (LC-MS grade), ammonium formate (LC-MS grade) and ammonium bicarbonate (LC-MS grade) were supplied by Honeywell Fluka (Seelze, Germany). Acetonitrile, methanol and water (LC-MS grade) were obtained from J.T Baker (Phillipsburg, NJ, USA). Citrate phosphate dextrose (CPD) human plasma was provided by Thai Red Cross Society (Bangkok, Thailand). Ethylenediaminetetraacetic acid (EDTA), Li-heparin and Na-heparin human plasma were acquired from six different healthy donors at Faculty of Tropical Medicine, Mahidol University (Bangkok, Thailand). Ethical approval for the method development and validation was given by the Ethics Committee of the Faculty of Tropical Medicine, Mahidol University, Bangkok, Thailand (approval certificate no. MUTM 2018-028-01). All healthy volunteers provided a written informed consent before blood donation.

### Sample preparation

***Preparation of standard and working solutions.*** Stock solutions of ceftriaxone (10 mg/ml) and cefotaxime (10 mg/ml) were prepared in water and methanol, respectively. The solutions were stored in cryo vials at -80°C. Working solutions of ceftriaxone were prepared by serial dilution of the stock solution in water and used for spiking of plasma samples. All solutions were allowed to equilibrate to room temperature before use. Haemolysed plasma was made by adding frozen and subsequently thawed whole blood to spiked plasma samples in an amount of 1.5% of total volume, which equals 2-2.5 g/l haemoglobin, resulting in moderately haemolysed plasma.

***Preparation of calibration standards and quality control samples.*** Calibration standards and quality control samples (QC) were prepared from two separate stock solutions to confirm the accuracy of the preparation. CPD human plasma was used to prepare calibration standards at concentrations of 1.01, 2.88, 8.21, 23.4, 66.7, and 200 μg/ml, including the lower limit of quantification (LLOQ: 1.01 μg/ml) and upper limit of quantification (ULOQ: 200 μg/ml), as well as over-curve dilution samples at 400 μg/ml. Quality control samples at 2.97, 24.1 and 155 μg/ml were prepared from a second stock solution. The final volume of working solution in plasma was less than 4% in all samples. Additional quality control samples were prepared with EDTA and heparin as anticoagulants.

***Extraction procedure.*** Sample extraction was performed by protein precipitation followed by phospholipid removal using Phree phospholipid removal cartridge (Phenomenex, CA, USA) on an automated liquid handler, Freedom Evo 200 platform (TECAN, Mannedorf, Switzerland). Plasma samples (100 μl) were manually aliquoted into a 96-well plate followed by protein precipitation using 400 µl internal standard solution (acetonitrile containing cefotaxime at 2 μg/ml) except for the double blank which used 400 µl acetonitrile. The plate was mixed at 1,000 rpm for 10 minutes on a Mixmate (Eppendorf, Hamburg, Germany) and centrifuged at 1,100 × g at 20°C for 5 minutes. The supernatant (300 μl) was loaded on the Phree phospholipid removal plate and vacuum was applied until the whole sample passed through the column. Finally, the extracted and cleaned sample was diluted with 500 μl water and mixed for 2 minutes at 1,000 rpm on a Mixmate and centrifuged at 1,100 × g for 2 minutes before injection.

### Instrument and chromatographic conditions

***Chromatography.*** The chromatographic separation was performed using a Dionex ultimate 3000 UHPLC (Thermo Scientific, CA, USA) consisting of a quaternary LC pump, a vacuum degasser, a temperature-controlled micro-well plate autosampler set at 10°C and a temperature-controlled column compartment set at 40°C. The LC systems were controlled by Chromeleon Chromatography Data System (CDS) 6.80 software (Thermo Scientific, CA, USA). The analytical column was an Agilent Zorbax Eclipse Plus C18 (100 × 2.1 mm; I.D. 3.5 μm (Agilent technologies, CA, USA) connected with pre-column C18 AJ0-7596, 4 × 2.0 mm (Phenomenex, CA, USA). The mobile phases consisted of (A) acetonitrile-ammonium formate (10 mM with 2% formic acid) (12.5:87.5 v/v), (B) acetonitrile-methanol (25:75 v/v) and (C) 20 mM ammonium bicarbonate. The mobile phase gradient was A: 0-2.0 min (0.4 ml/min), B:C (5:95 v/v): 2.1-4.1 min (0.6 ml/min), B:C (90:10 v/v): 4.2-6.2 min (0.6 ml/min), and A: 6.3-10.0 min (0.4 ml/min), resulting in a total run time of 10 min. A sample volume of 2 μl was injected into the LC system.

***Mass spectrometry.*** An API 5000 triple quadrupole mass spectrometer (SCIEX, MA, USA) was used for the detection and quantification. Data acquisition and analysis were performed using the Analyst® 1.7 software (SCIEX, MA, USA). The TurboV ionisation source (TIS) interface was operated in the positive ion mode with a drying temperature of 500°C. The interface voltage was set to 5.5 kV. The curtain, nebulizer, TIS gas pressure and declustering potential were set at 35, 50, 55 psi and 90 V, respectively. The selected reaction monitoring (SRM) was used to detect and quantify the precursor-product ion transitions *m/z* 555.0→396.1 for ceftriaxone and 456.0→324.0 for cefotaxime with a collision energy of 20 and 39 V, respectively.

### Method validation

The method was validated according to the US Food and Drug Administration (FDA) guidelines (2001) on bioanalytical method validation ^17^. Accuracy and precision were determined by analysing five replicates of five concentrations (1.01, 2.97, 24.1, 155, 200 μg/ml) from four separate runs. The over-curve samples of 400 μg/ml were diluted with blank plasma (1:10) to evaluate dilution integrity. Accuracy was calculated by comparing the mean measured concentration to the nominal concentration at each QC level. Precision of the assay was evaluated by using analysis of variance (ANOVA) via the Analysis ToolPak add-in to Microsoft Excel 2016 (Microsoft, Redmond, WA, USA) and reported as the relative standard deviation (%RSD). Acceptance criteria for precision and accuracy are ±15%, except for LLOQ where ±20% is acceptable.

***Linearity, selectivity and recovery.*** Linearity was evaluated by individually analysing the calibration standards from four separate runs. The regression model that resulted in the best accuracy of back-calculated concentrations of the calibration curves and QC samples was selected as the most appropriate regression model. Linear regression models, non-weighted and with weighting (1/ *x* and 1/ *x* ^2^), as well as quadratic model with 1/ *x* weighting, were evaluated. Acceptance criteria for linearity are that 75% of non-zero calibrators should be within ±15%, except for LLOQ where ±20% is acceptable.

Selectivity was evaluated by injecting blank extracted samples and potentially interfering drugs during a regular analysis run and then, as post-column infusion (10 µl/min) of 1 µg/ml ceftriaxone and 1 µg/ml cefotaxime (internal standard) in water to confirm that there was no signal that potentially could interfere with the drug identification and measurement. Six blank heparin plasma samples from six different blood donors and samples containing different anticoagulants (EDTA, CPD, Li-heparin and haemolysed Na-heparin) were used for the analysis. Potentially interfering drugs (i.e. acetaminophen, doxycycline and azithromycin, at a concentration of 100 ng/ml in methanol-water 20:80 v/v equivalent to a pre-extraction sample concentration of 1.5 µg/ml) were also evaluated. The occurrence of a peak response at the retention time of the analyte or internal standard indicates an interference and would require further investigation. Acceptance criteria for selectivity are that interference should be less than 20% of LLOQ and less than 5% of the internal standard response.

Recovery was determined by comparing two sets of samples. One set was spiked with ceftriaxone and internal standard before extraction (i.e. pre-spiked) and extracted as described in the method, including internal standard. However, to minimize variations as the Phree plate will retain some extraction liquid, a fixed volume of 150 µl extracted Phree eluate was taken and mixed with 500 µl water. The second set was extracted blank plasma with post-extract addition of ceftriaxone and internal standard, where 150 µl extracted blank plasma Phree eluate were taken and mixed with 350 µl water and 150 µl spiked water solution containing the same nominal concentration of ceftriaxone and internal standard as set 1. Thus, both sets contained the same volume ratio of extracted biological sample, acetonitrile and water. Recovery was determined by comparing the peak response of individual pre-spiked samples of set 1 to the average peak response of post-extract addition samples in set 2. Five replicates of each concentration at 2.97, 24.1 and 155 μg/ml were evaluated.

***carry-over testing.*** The carry-over effect was investigated by injecting three replicates of blank samples after five injections of samples at ULOQ concentrations. To verify that this carry-over would not accumulate over time, carry-over was therefore tested in all 4 precision and accuracy batches and the carry-over set was positioned to run after approximately 50 sample injections had passed from the precision and accuracy batch. The presence of a signal greater than 20% of the LLOQ or 5% of the internal standard indicates carry-over.

***Matrix effects.*** Matrix effect was assessed by both post-column infusion (qualitative visualization) ^18, 19^ and post-extraction addition (quantitative evaluation), in a simplified approach described by Matuszewski *et al.* ^20^. The matrix factor was calculated by comparing the peak response of extracted blank plasma using post-extract addition of ceftriaxone and internal standard to the average peak response of the analytes in neat matrix free reference solution at the same nominal concentrations. As in the recovery test, the same volume ratio of acetonitrile and extracted sample/water was maintained. Two concentrations (low and high) at 2.97 and 155 μg/ml were evaluated. Heparin plasma from six different donors was used for the analysis as well as haemolysed plasma. Different anticoagulants EDTA, CPD, Li-heparin were evaluated. The acceptance criteria for matrix effects was achieved when the RSD of the internal standard normalised matrix factor calculated from the 6 lots of donor matrix was below ±15%.

***Stability.*** Spiked plasma stored at ambient temperature and at 4°C for 48 h was used to evaluate short-term stability. Long-term stability of spiked samples at -80°C was evaluated after 7 months. Freeze-thaw stability was evaluated for plasma samples and haemolysed plasma samples for five cycles. The samples were stored at -80°C for 24 h followed by unassisted thawing at room temperature for 2–3 h and subsequent re-freezing at -80°C. The stability of precipitated samples stored at ambient temperature (about 23°C) for 4 h was also evaluated. The stability of extracted samples in the LC autosampler kept at 10°C was evaluated by re-injecting the calibrators and QC samples 65 h after initial injection. The acceptance criteria for stability was achieved when the RSD of stability samples was below ±15%, and the accuracy of mean concentrations was within ±15% of nominal concentration.

## Results and discussion

The calibration range of 1.01-200 μg/ml was based on pharmacokinetic data from previously published studies ^6, 8, 21^, taking into account the sensitivity and linearity of the MS instrument. Reported population mean peak levels of ceftriaxone was reported to be below 200 μg/ml after a standard 2-g daily dose in critically ill patients with sepsis ^8^. There is a possibility that some clinical samples have higher concentrations of ceftriaxone than covered by the calibration range. However, to maintain the ability to quantify these high-concentration samples, sample dilution integrity needs to been shown. An over-curve sample concentration of 400 μg/ml was evaluated for dilution integrity and demonstrated that such samples can be diluted and quantified using the developed method. Mean plasma concentrations, 24 h after administration of ceftriaxone, were reported to be 5.3, 9.3 and 15.1 μg/ml after 0.5-g, 1-g, and 2-g of intravenous dose, suggesting adequate sensitivity to quantify the drug in patients to evaluate the pharmacokinetic properties ^6^.

### Sample preparation and extraction

Various extraction solvents were evaluated for protein precipitation. Adding an acid, such as acetic acid or formic acid, often improves the precipitation of proteins and can improve recovery. However, acidic storage conditions affected the stability of ceftriaxone and degradation was observed. Neat acetonitrile and methanol both worked well as protein precipitation solvents. The results indicated that acetonitrile yielded lower ceftriaxone extraction recovery than methanol. However, methanol likely extracted more interfering components from plasma samples which gave more matrix effects compared to acetonitrile. To improve the sample clean-up further, three different phospholipid removal filtration plates were evaluated; HybridSPE (Supelco, PA, USA), Ostro (Water, MA, USA) and the Phree plate. The HybridSPE plate retained ceftriaxone, giving very low recovery yield. Both Phree and Ostro phospholipid removal plates showed similar performance with a recovery difference of less than 10% compared to only protein precipitation. The Phree plate was selected based on price and performance.

### Instrumentation and chromatographic condition

Peak tailing of ceftriaxone has been observed and reported in the literature previously ^14, 15, 22^. Various chromatographic columns (i.e. C18, C6-phenyl, CN and amide stationary phases) and mobile phases were screened in this study, but peak tailing of ceftriaxone could not be eliminated completely. Best peak shape was obtained with the C18 end capped column from Agilent Zorbax Eclipse Plus and used throughout validation experiments.

To evaluate the effectiveness of the sample clean-up and how much phospholipids passes through the LC column, fragment ions m/z 104 and 184 was monitored as described by Ismaiel *et al,* ^23^. Protein precipitation resulted in a significant amount of phospholipids left in the sample while phospholipid removal plates resulted in a clean sample with very low amount of phospholipids left in the sample. No phospholipid interference was seen at the retention time of analyte or IS. Residues of strongly retained phospholipids could be eluted by utilising a LC-washout gradient of acetonitrile-methanol (25:75 v/v) preventing accumulation on the LC-column or interference of late eluting phospholipids in subsequent injections. Phospholipid removal plates also filtrated proteins and particles, and are particular useful for clinical studies with a large number of samples to process (i.e. less problems and downtime).

**Figure 2.**
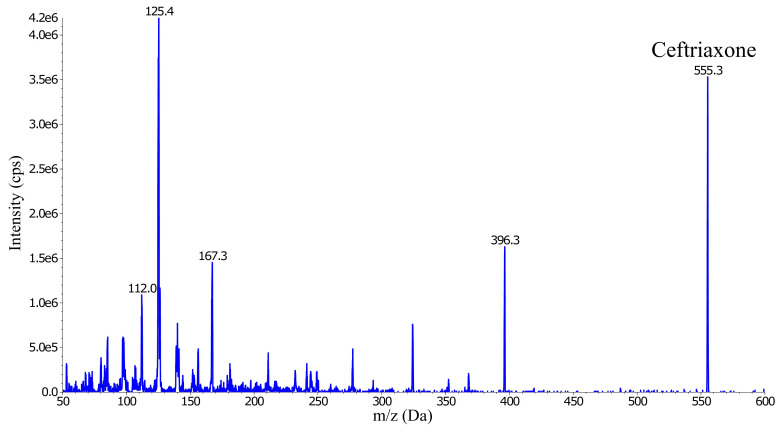
Collision energy scan and fragmentation product ions of ceftriaxone (555.3 *m/z*).

The ESI MS was operated in the positive ion mode and generated several abundant ceftriaxone fragment ions; m/z 396.3, 324.1, 167.3, 125.4 and 112.0 ( Figure 2). Three of these fragment ions ( *m*/ *z* 396.3, 167.3 and 125.4) were evaluated for signal intensity and selectivity, and for any signs of interference. The precursor-product ion transition *m/z* 555.0→396.1 was selected as the quantification trace because it showed approximately twice the intensity compared to the other two fragments.

Deuterium-labelled ceftriaxone (D _3_) was evaluated in the method development phase as an internal standard. In positive ion mode it generated two fragment ions containing deuterium. The two fragment ions ( *m*/ *z* 399.0 and 327.0) were evaluated for signal intensity and selectivity, and for any signs of interference. Unfortunately, ceftriaxone interfered with the ceftriaxone-D _3_ signal in the LC-MS/MS instrument. This could be explained by the isotopic distribution of ceftriaxone, were its third isotope have the same mass as ceftriaxone-D _3_ and hence cause interference ^24, 25^. The signal contribution from a ULOQ sample was about 40% of the ceftriaxone-D _3_ internal standard signal (concentration of 1 µg/ml). Lowering the calibration range (ULOQ) and increasing the D _3_-internal standard concentration would still produce a signal contribution to D _3_-internal standard with more than 5%, thus over the acceptance limit for signal interference to internal standard. There are other stable isotope internal standards, but these could not be evaluated due to time and funding restrictions. Thus, a substitute internal standard (cefotaxime) was chosen, which belongs to the same class of antibiotic as ceftriaxone but the two drugs are not administered together.

### Validation

Accuracy and precision were evaluated by an ANOVA approach and all concentration levels were within the acceptance criteria, including the over-curve dilution integrity samples ( Table 1). Alternative anticoagulants (EDTA, Na-heparin, Li-heparin) were evaluated at low and high QC levels and were within the acceptance criteria ( Table 2). Raw data are available on Figshare ^26^.

**Table 1.** Accuracy and precision of ceftriaxone determination. The method was validated by analysing five replicate samples of each concentration and repeated over four days. Accuracy and precision must not exceed 15% for each concentration, except for the LLOQ that should not deviate by more than 20%.

Value	Nominal conc. (μg/ml)	Intra-assay precision (%RSD)	Inter-assay precision (%RSD)	Total-assay precision (%RSD)	Accuracy (%)
LLOQ	1.01	4.31	4.18	4.29	0.50
QC 1	2.97	4.22	3.95	4.18	-13.6
QC 2	24.1	3.94	5.57	4.24	-8.90
QC 3	155	2.21	8.68	4.00	-13.0
ULOQ	200	3.29	8.71	4.59	2.80
Over-curve	400	3.59	9.29	4.95	-3.50

LLOQ, lower limit of quantification; QC, quality control; ULOQ, upper limit of quantification; Over-curve, i.e. sample dilution 10 times; RSD, relative standard deviation.

**Table 2.** Accuracy and precision of ceftriaxone in different anticoagulants. The method was validated by analysing five replicate samples of each concentration and repeated over four days. Accuracy and precision must not exceed 15% for each concentration. However, accuracy is not reported since the QC samples were compared against a calibration curve using CPD plasma and the recovery difference would bias the accuracy result.

Anticoagulant	Nominal conc. (μg/ml)	Intra-assay precision (%RSD)	Inter-assay precision (%RSD)	Total-assay precision (%RSD)
EDTA, QC 1	2.97	5.52	5.56	5.54
Na-Heparin, QC 1	2.97	7.53	13.5	8.75
Li-Heparin, QC 1	2.97	7.35	9.00	7.64
EDTA, QC 3	155	3.81	4.76	3.98
Na-Heparin, QC 3	155	4.10	10.5	5.62
Li-Heparin, QC 3	155	3.77	5.40	4.07

QC, Quality Control; RSD, Relative Standard Deviation.

***Linearity, selectivity and recovery.*** The calibration curve was evaluated for linearity by different calibration models. The model that described the best concentration-response relationship was a linear regression with 1/ *x* ^2^ weighting, resulting in an accuracy of back-calculated concentration ranging from 92.1–104%. For selectivity, no interfering peaks were present in the blank plasma injections from the six different donors. Moreover, injection of possible concomitant drugs (i.e. acetaminophen, azithromycin and doxycycline) did not produce any interference. Blank plasma samples with CPD, EDTA, sodium heparin, lithium heparin and a sodium heparin sample with haemolysis were also evaluated. None of the anticoagulants or the haemolysis sample produced any interference.

The Phree plate and heparin plasma was used for determining ceftriaxone and internal standard recovery. The results showed a recovery of 30–35% for ceftriaxone while the internal standard achieved 98-100% recovery. There was a clear recovery difference for ceftriaxone using different anticoagulants, where CPD plasma generally achieved 10–15% higher recovery compared to heparin and EDTA about 5–10% higher compared to heparin. Using the same anticoagulant in both calibrators and study samples is therefore important to avoid a bias in the result.

**Figure 3.**
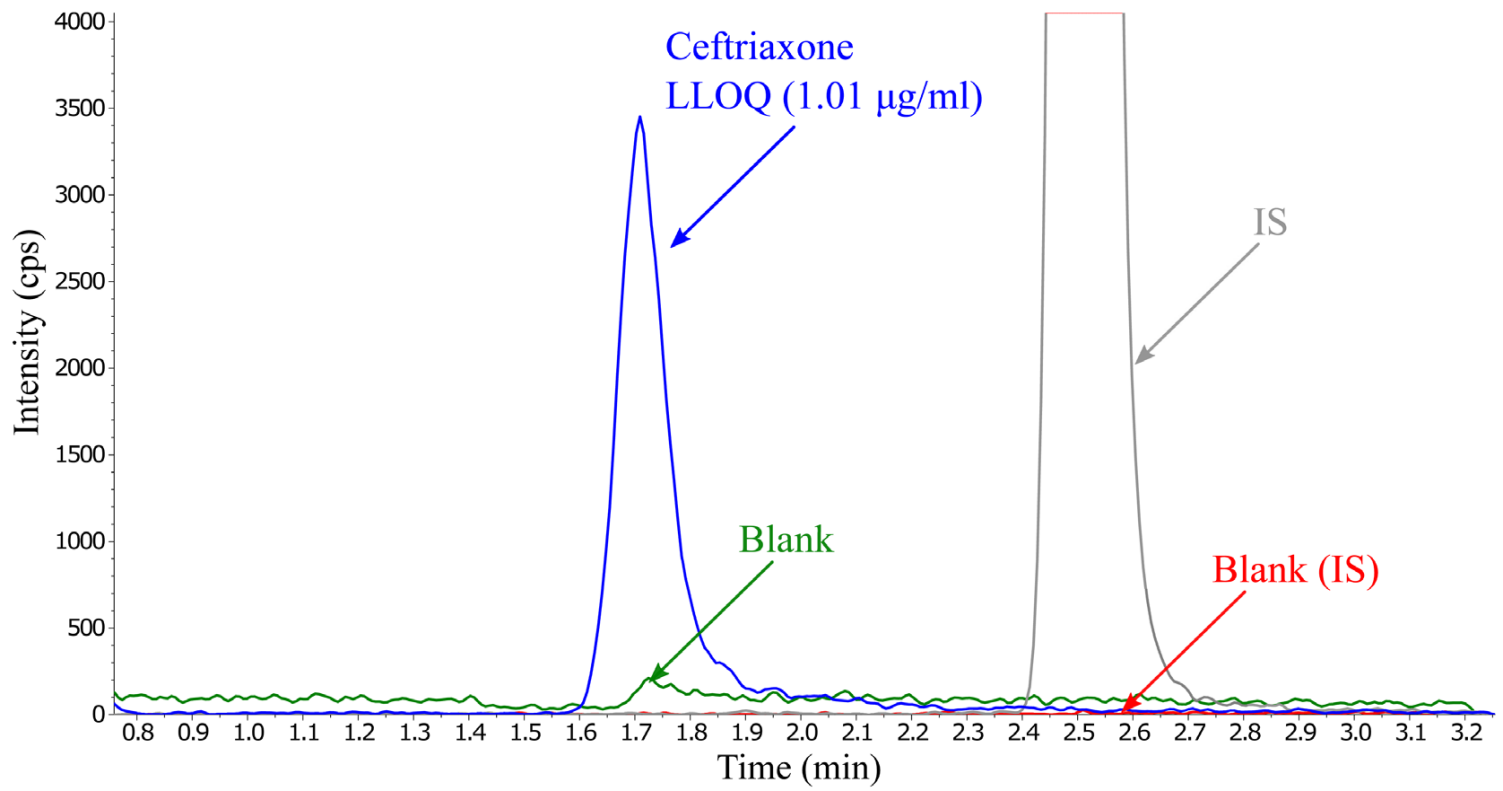
Overlay of ceftriaxone at LLOQ concentration containing internal standard (2 µg/ml) and the first blank injection after injecting five ULOQ samples, presenting no significant carry-over.

***carry-over testing.*** Carry-over was a problem and difficult to eliminate. Initially an Agilent 1260 infinity system (Agilent technologies, CA, USA) was used and extensive testing with advanced needle wash programming and rotor changes was performed without being able to eliminate the carry-over. Later, a Dionex ultimate 3000 UHPLC was used, switching stainless steel to biocompatible tubing and introducing injection rotor switching during run did not prevent the carry-over issue. However, the carry-over did reduce over-time as the mobile phase flowed through the system and was eliminated given enough time between injections. To reduce the waiting time between injections, different washout solvents and solution mixes were tested. Carry-over was minimized by adding a washout step, using ammonium bicarbonate, in combination with a total run-time of 10 minutes ( Figure 3).

***Matrix effect.*** Matrix effect evaluation during post-column infusion did not show any increase or drop in ceftriaxone or internal standard signals. Injection of possible concomitant drugs or plasma with different anticoagulants, including haemolysis-plasma, did not show any increase or decrease in the signal. The quantitative matrix effect test resulted in a 27% signal enhancement of extracted blank CPD plasma with post-extract addition of ceftriaxone at a concentration of 2.97 μg/ml concentration level. This signal enhancement could not be fully compensated by the internal standard. The internal standard normalised matrix factor still showed a 24% enhancement of the CPD plasma signal. However, all other samples showed a normalised matrix factor within ± 15%. The six different sources of plasma from donors A-F collected in the same sodium heparin anticoagulant showed an average normalized matrix factor of 108% ± 5.7% SD and an RSD of 5.2%. This suggests that the precision of the method is not affected by different lots of plasma using the same anticoagulant when compared to the method precision in Table 2. The high concentration level of 155 μg/ml showed suppression of the signal but stayed within acceptable limits ( Table 3). Matrix effects have also been reported by other authors, affecting only the lowest concentrations. Common features for all methods is the use of protein precipitation using either methanol or acetonitrile and all methods used C18 LC-columns for separation ^14, 15, 27, 28^. LC-MS separation and detection might not be able to avoid matrix effects ^29^, but Decosterd *et al.* ^30^, reported that even with matrix effects at 165%, the use of ceftriaxone-13CD _3_ stable isotope internal standard compensated fully for this matrix effect. This resulted in a normalized matrix factor of 101.7%, demonstrating the importance of using a suitable internal standard. The internal standard in this work did not show any matrix effects and can therefore do little to compensate for matrix effects affecting ceftriaxone. A more suitable stable isotope internal standard would have been desirable in this case, and would most likely have experienced the same matrix effect as ceftriaxone and therefore compensated for any potential differences in the signal ^30^.

**Table 3.** Matrix effects from different donors in heparin plasma and different anticoagulants. Donors A to F are individual donors collected using sodium heparin as anticoagulant. Other anticoagulants were collected from individual donors and are not from the same source.

Concentration	Donor						EDTA	CPD	Li-Hep	Na-Hep haemolysis
	A	B	C	D	E	F				
QC 1, 2.97 µg/ml	1.12	1.18	1.10	1.03	1.07	1.04	1.10	1.27	1.10	1.17
QC 3, 155 µg/ml	0.93	0.87	0.93	0.91	0.93	0.88	0.93	0.96	0.90	0.91
IS for QC 1, 2 µg/ml	0.99	1.03	1.00	1.01	1.01	1.01	1.03	1.02	1.03	1.05
IS for QC 3, 2 µg/ml	1.01	1.05	1.03	1.05	1.01	1.03	1.04	1.04	1.03	1.05
Normalised QC1/IS	1.13	1.15	1.10	1.02	1.06	1.02	1.06	1.24	1.07	1.12
Normalised QC3/IS	0.93	0.84	0.90	0.87	0.92	0.86	0.89	0.92	0.88	0.87

Hep, Heparin; QC, Quality Control; IS, internal standard.

***Stability.*** The stability samples were quantified using a calibration curve in CPD plasma. Stability samples in CPD plasma were compared to the average measured concentration of CPD QC samples added in the same run. The CPD calibration curve was also used to quantify heparin and EDTA stability samples due to limited supply of volunteer donor blood. However, since EDTA and heparin have different recovery from plasma compared to CPD, a direct comparison would be biased. Thus, stability samples were instead compared with the average measured concentration of the precision and accuracy of each anticoagulant. Short-term stability for up to 24 h at ambient temperature (about 23°C) and 4°C for ceftriaxone was confirmed in all anticoagulants and for CPD plasma up to 48 h. Long-term stability at -80°C was evaluated after 7 months (224 days) and showed good stability for all anticoagulants. QC samples in all anticoagulants presented good stability after freeze-thaw over five cycles, including plasma with moderate haemolysis. Protein precipitated samples also showed good stability when stored at ambient temperature (about 23°C) for 4 h prior to transferring the supernatant to the Phree phospholipid removal plate ( Table 4). Extracted samples in the LC autosampler, up to 65 h, showed less than 10% variation in QC concentrations if the full set of calibrators and QC was re-injected. However, comparing the original injection with the 65-h injection did show a loss of about 20%; however, the change is equal over the whole concentration range and will not be noticed if the full set of calibrators and QC are re-injected.

**Table 4.** Stability of ceftriaxone in plasma under different conditions. Due to the recovery difference between anticoagulants, EDTA, Na-heparin and Li-heparin are compared to the average concentration of the four precision and accuracy batches for each anticoagulant and are presented as percentages.

QC1, 2.97 µg/ml	RT 24 hrs	RT 48 hrs	4°C 24 hrs	4°C 48 hrs	F/T cycle 3	F/T cycle 5	Precipitated 4hrs in RT	-80°C 224 days
CPD	106	100	102	103	97.7	94.0	94.2	103
CPD haemolysis	-	-	-	-	-	88.4	99.3	-
EDTA	105	-	113	-	103	103	98.0	95.5
Na-Hep	103	-	109	-	100	98.7	96.8	93.7
Na-Hep haemolysis	-	-	-	-	91.3	97.6	91.9	-
Li-Hep	105	-	98.3	-	95.7	99.5	103	96.0
QC3, 155 µg/ml	RT 24 hrs	RT 48 hrs	4°C 24 hrs	4°C 48 hrs	F/T cycle 3	F/T cycle 5	Precipitated 4hrs in RT	-80°C 224 days
CPD	99.8	99.6	101	103	99.1	95.0	101	109
CPD haemolysis	-	-	-	-	-	98.6	94.3	-
EDTA	105	-	104	-	97.9	95.5	90.9	92.8
Na-Hep	103	-	106	-	97.5	93.7	88.2	94.5
Na-Hep haemolysis	-	-	-	-	90.4	88.5	88.8	-
Li-Hep	101	-	108	-	96.1	94.2	91.4	98.8

Hep, heparin; RT, ambient room temperature (about 23°C), F/T, freeze and thaw, “-“, not available.

## Conclusion

The use of LC-MS/MS resulted in higher sensitivity and selectivity than HPLC-UV. The developed method requires only a small volume of plasma (100 μl) and will allow for pharmacokinetic studies in children and other groups with limited sampling capabilities. However, there might still be a limitation for very small children, infants and neonates where only a very small amount of blood can be obtained from venepuncture or capillary sampling. Moreover, the incorporation of phospholipid removal techniques during sample preparation reduced particles and matrix interferences that could otherwise risk clogging the system and/or accumulate on the column. This sample preparation technique should preserve the MS instrument and column over time, enabling long-term usage without interruptions. Carry-over problems were solved by modifying the LC-gradient program by including an additional washout sequence. However, the spiked QC samples in EDTA and heparin plasma showed lower recovery than CPD. Thus, it is important to use the same anticoagulant in calibration curves and clinical samples for analysis. Spiked plasma samples showed good stability in various conditions over a short term and the extracted samples can be re-injected from the LC autosampler up to 65 h after extraction.

## Data availability

Figshare: Supplementary files ceftriaxone plasma. https://doi.org/10.6084/m9.figshare.7775819.v1 ^26^.

The following underlying data are available:

- Long-term stability 224 days.txt (Quantification data for long-term stability calculations of ceftriaxone in CPD, EDTA, Na-heparin and Li-heparin plasma)
- Precision and Accuracy run 1.txt (Quantification data for run 1 out of 4, for the accuracy and precision used in ANOVA calculations)
- Precision and Accuracy run 2.txt (Quantification data for run 2 out of 4, for the accuracy and precision used in ANOVA calculations)
- Precision and Accuracy run 3.txt (Quantification data for run 3 out of 4, for the accuracy and precision used in ANOVA calculations]
- Precision and Accuracy run 4.txt [Quantification data for run 4 out of 4, for the accuracy and precision used in ANOVA calculations)
- Recovery and matrix effects.txt (Peak areas of extracted QC samples, blank plasma post spiked and reference in neat solution for recovery and matrix effect calculations).
- Stability 4 hrs Haemolysis and Precipitation at RT.txt (Quantification data for the stability of precipitated samples in clear plasma and haemolysed plasma in different anticoagulants, stored 4 h in room temperature before transferring supernatant to Phree plate).
- Stability Freeze and Thaw.txt (Quantification data for testing repeated freeze and thaw stability of ceftriaxone in plasma using different anticoagulants including haemolysed plasma).
- Stability LC-stability over 65 hrs.txt (Quantification data testing ceftriaxone stability, comparing the difference in quantified concentration from original injected samples re-injection 65 h later).
- Stability RT and 4C 4hrs-48hrs.txt (Quantification data testing ceftriaxone stability in plasma with different anticoagulants stored in room temperature or in 4°C for 24 h (CPD tested up to 48 h)).

Data are available under the terms of the Creative Commons Zero "No rights reserved" data waiver (CC0 1.0 Public domain dedication).
