# Supplementary material for: Determination of ceftriaxone in human plasma using liquid chromatography–tandem mass spectrometry
Source: Wellcome Open Res. 2022 May 31;4:47. Originally published 2019 Mar 8. [Version 3] doi: 10.12688/wellcomeopenres.15141.3 (PMC9379334; doi:10.12688/wellcomeopenres.15141.3)
Supplement: Peer review history

# Peer review: Reviewer response for version 3

**DOI:** 10.21956/wellcomeopenres.19879.r51333

**Published:** 2022-08-15

**Authors:** Daan Touw

**Affiliations:** 1 Department of Clinical Pharmacy and Pharmacology, University Medical Center Groningen, University of Groningen, Groningen, The Netherlands

The authors describe the development and validation of an LC-MSMS assay for ceftriaxone in human plasma. Analysis of beta-lactam antibiotics such as ceftriaxone is relevant from a patient perspective. The pharmacodynamic driver for ceftriaxone efficacy is the time the concentration of the free drug is above the minimal inhibitory concentration of the targeted microorganism. Especially in critically ill patients, pharmacokinetics of beta-lactam antibiotics may be altered and dosing guided by the measurement of plasma concentration may be of great help and has demonstrated clinically useful.

 The authors follow the guidelines issued by the FDA and the EMA for bioanalytical method validation.

 The research is well conducted and scientifically sound. Data are available and the method is well described to replicate the method in other laboratories.

 Presently, only the total concentration of the drug is analysed. A next step would be the analysis of the free drug (not protein bound plasma fraction).

Is the rationale for developing the new method (or application) clearly explained?

Yes

Is the description of the method technically sound?

Yes

Are the conclusions about the method and its performance adequately supported by the findings presented in the article?

Yes

If any results are presented, are all the source data underlying the results available to ensure full reproducibility?

Yes

Are sufficient details provided to allow replication of the method development and its use by others?

Yes

Reviewer Expertise:

Analytical method development, therapeutic drug monitoring, clinical toxicology

I confirm that I have read this submission and believe that I have an appropriate level of expertise to confirm that it is of an acceptable scientific standard.

# Peer review: Reviewer response for version 2

**DOI:** 10.21956/wellcomeopenres.18966.r47994

**Published:** 2022-02-07

**Authors:** Stanislas Grassin-Delyle

**Affiliations:** 1 Department of Health Biotechnology, UFR Simone Veil - Santé, Université Versailles Saint Quentin, Versailles, France; 2 Department of Respiratory Diseases, Foch Hospital, Suresnes, France

Wongchang and colleagues describe a method for the quantification of ceftriaxone in human plasma by liquid chromatography-mass spectrometry.

 The introduction clearly documents the state of the art and the need for specific and sensitive methods for the quantification of ceftriaxone in human plasma. It appears that other methods have been published since the initial submission of the article and would be worth mentioning in the introduction e.g. Meenks S *et al*., (2021) ^1^and Herrera-Hidalgo L *et al*., (2021) ^2^. In addition, the possible advantages of the described method over existing methods should be better highlighted in the Discussion.

**Chromatographic separation:**

 As I understand it, the mobile phase consists of 2 binary mixtures plus a third phase of ammonium bicarbonate, with the application of a composition gradient and a flow rate gradient during the 10-minute run. This gradient appears complex and the simultaneous use of acetonitrile and methanol as organic modifiers is unconventional, as is the use of both ammonium formate and ammonium bicarbonate. The pH values of the different phases should be clarified and the use of such a complex mixture justified, especially as it appears that a simpler phase can be used with a shorter run time (Herrera-Hidalgo L *et al*., 2021 ^2^).

**Method validation:**

 The authors refer to the 2001 FDA guideline while an updated version was published in 2018. The authors should clarify which parameters are assessed according to the guideline, e.g. stability of stock solutions or incurred sample reanalysis are not reported. Quantitative values could be presented for recovery for ceftriaxone and internal standard, comparison of different reagents used for sample preparation (acids, solvents, phospholipid precipitation plates), carry-over.

**Matrix effect:**

 The results between the two methods used (post-column infusion and injection of plasma spiked before or after the sample preparation procedure) and the nature of the anticoagulants tested in each procedure should be better explained. Mean values and their dispersion are not shown in Table 3. Why do individual values appear for 6 donors with sodium heparin in Table 3, but only one value for the other anticoagulants? Which anticoagulants were tested with the post-column infusion method? With what concentration of ceftriaxone? The presentation of the plots obtained with the first method could help interpretation and the exact procedure used to calculate the matrix effect value detailed in full. The results presented as supplementary data in the file "Recovery and matrix effects.txt" do not help to understand how the value of +27% was observed, nor how this effect would be compensated by the internal standard.

 What does the sentence "The high concentration level of 155 μg/ml showed suppression of the signal but stayed within acceptable limits (Table 3)." mean? If the value is within acceptable limits, then there is no suppression of the signal.

**Results with clinical samples:**

 For publications reporting the development of analytical methods, demonstration that the method works correctly on real clinical samples is highly desirable.

**Minor comments:**

- Abstract: ceftriaxone is a third generation cephalosporin (not antibiotic).
- Internal standard is not "quantified.
- Preparation of stock solutions: it should be specified whether the 10 mg/ml of stock solutions refer to the sodium salts or base drug.
- "Sample preparation" may be preferred to "sample extraction" as the preparation procedure consists in protein precipitation and phospholipid removal.

Is the rationale for developing the new method (or application) clearly explained?

Yes

Is the description of the method technically sound?

Partly

Are the conclusions about the method and its performance adequately supported by the findings presented in the article?

Partly

If any results are presented, are all the source data underlying the results available to ensure full reproducibility?

Partly

Are sufficient details provided to allow replication of the method development and its use by others?

Partly

Reviewer Expertise:

Mass spectrometry; metabolomics; pharmacokinetics

I confirm that I have read this submission and believe that I have an appropriate level of expertise to confirm that it is of an acceptable scientific standard, however I have significant reservations, as outlined above.

## References

[1] : Liquid Chromatography-Tandem Mass Spectrometry to Monitor Unbound and Total Ceftriaxone in Serum of Critically Ill Patients. *Curr Rev Clin Exp Pharmacol* .2021;16(4) : 10.2174/1574884715666201228115150 341-349 10.2174/1574884715666201228115150 33371859

[2] : Ceftriaxone pharmacokinetics by a sensitive and simple LC-MS/MS method: Development and application. *J Pharm Biomed Anal* .2020;189: 10.1016/j.jpba.2020.113484 113484 10.1016/j.jpba.2020.113484 32736331

# Response

**Published:** 2022-05-27

**Authors:** Daniel Blessborn

**Affiliations:** Mahidol-Oxford Tropical Medicine Research Unit, Thailand

Wongchang and colleagues describe a method for the quantification of ceftriaxone in human plasma by liquid chromatography-mass spectrometry.

 The introduction clearly documents the state of the art and the need for specific and sensitive methods for the quantification of ceftriaxone in human plasma. It appears that other methods have been published since the initial submission of the article and would be worth mentioning in the introduction e.g. Meenks S *et al*., (2021) ^1^ and Herrera-Hidalgo L *et al*., (2021) ^2^ . In addition, the possible advantages of the described method over existing methods should be better highlighted in the Discussion.

***Response:***

*Thank you for these suggestions. We believe that the introduction should describe a short overview of what have been published prior to the validation of the assay. In our case, validation was completed in mid-2018 and the method was published in early 2019, and we think it would be somewhat misleading if methods published after 2019 were to be added to the introduction. However, we have added the latest developments and compared these with our result. As suggested, we have added a section on matrix effects and stability in the discussion, as well as the publications of Meenks S et al., (2021) and Herrera-Hidalgo L et al., (2021).*

**Chromatographic separation:**

 As I understand it, the mobile phase consists of 2 binary mixtures plus a third phase of ammonium bicarbonate, with the application of a composition gradient and a flow rate gradient during the 10-minute run. This gradient appears complex and the simultaneous use of acetonitrile and methanol as organic modifiers is unconventional, as is the use of both ammonium formate and ammonium bicarbonate. The pH values of the different phases should be clarified and the use of such a complex mixture justified, especially as it appears that a simpler phase can be used with a shorter run time (Herrera-Hidalgo L *et al*., 2021 ^2^ ).

***Response:***

*The acetonitrile/methanol mixture was initially explained in the result and discussion section “instrument and chromatographic conditions”. In the revised manuscript, we clarified and explained how both acetonitrile/methanol and ammonium bicarbonate were used to reduce carry-over and that the increased washout flow rate reduced the run time to 10 minutes. The pH of ammonium bicarbonate has been added as suggested.*

*The article by Herrera-Hidalgo L et al., (2021) had indeed a simpler mobile phase and shorter run time, but their method also showed lower sensitivity compared to our assay (i.e. lower limit of quantification of 3.00 vs 1.01 µg/ml). They also reported matrix effect of QC1-124%, QC2-112% and QC3-123%, a carry-over but kept it below 20%, and a short LC-stability of only 4 hrs. In fact, most of the published methods have some sort of drawbacks, e.g. matrix effects, carry-over effects, long run times, limited calibration ranges, low sensitivity and/or bad peak symmetry etc. These aspects makes the method development of ceftriaxone more challenging and we have added this in the discussion section.*

**Method validation:**

 The authors refer to the 2001 FDA guideline while an updated version was published in 2018. The authors should clarify which parameters are assessed according to the guideline, e.g. stability of stock solutions or incurred sample reanalysis are not reported. Quantitative values could be presented for recovery for ceftriaxone and internal standard, comparison of different reagents used for sample preparation (acids, solvents, phospholipid precipitation plates), carry-over.

***Response:***

*As the reviewer suggested, we have added the stability data of stock solutions and working solutions as well as a table showing the recovery of ceftriaxone and internal standard in heparin plasma.*

*We referred to FDA2001 guideline as the validation took place at the same time the FDA2018 was being published. However, the validation presented here are also compliant with the EMA2012 guideline that includes carry-over testing and dilution integrity tests etc. In fact, the EMA-2012 and FDA-2018 are very similar. We have added EMA2012 in the reference as well and an open access article reference that explain the minor differences between these guidelines.*

*As for “comparison of different reagents used for sample preparation (acids, solvents, phospholipid precipitation plates), carry-over.” These tests were performed during different stages of the method development using different concentrations and also in some cases a different model of the MS instrument. It would be difficult to present all the different parameters in one table without extensive explanations of differences and changes between each experiment. We believe that a simple explanation and overall result, as described in the article, would be most informative and hope that this is acceptable.*

**Matrix effect:**

 The results between the two methods used (post-column infusion and injection of plasma spiked before or after the sample preparation procedure) and the nature of the anticoagulants tested in each procedure should be better explained. Mean values and their dispersion are not shown in Table 3. Why do individual values appear for 6 donors with sodium heparin in Table 3, but only one value for the other anticoagulants? Which anticoagulants were tested with the post-column infusion method? With what concentration of ceftriaxone? The presentation of the plots obtained with the first method could help interpretation and the exact procedure used to calculate the matrix effect value detailed in full. The results presented as supplementary data in the file "Recovery and matrix effects.txt" do not help to understand how the value of +27% was observed, nor how this effect would be compensated by the internal standard.

***Response:***

*We have revised this section to clarify what samples and anticoagulants were included in the post-column infusion evaluation as well as the concentration of the post-column infusion solution. A new figure was added in the revised manuscript, showing the post column infusion as overlay of Donor A and B in sodium heparin plasma and a CPD as anticoagulant to show that they did not differ visually.*

*Mean values and their dispersion have also been added to the matrix effect table as suggested. The matrix effect table have also been split in two separate tables as one table is for the 6 donors with sodium heparin and a separate table for the additional tests of different anticoagulants. FDA and EMA bioanalytical guidelines specify that 6 individual donors have to be screened for selectivity and that was done using sodium heparin as anticoagulant.*

*For the question “supplementary data in the file "Recovery and matrix effects.txt" and how the value of +27% was observed”. To calculate the matrix effect, peak area of “Blank X no IS post addition QC 1 CPD” was divided with the average peak areas of “Neat Reference QC1 + IS”, and that will result in 1.27 (+27%).*

 What does the sentence "The high concentration level of 155 μg/ml showed suppression of the signal but stayed within acceptable limits (Table 3)." mean? If the value is within acceptable limits, then there is no suppression of the signal.

***Response:***

*The reviewer is correct, if within acceptable limits, there is no suppression of the signal. The sentence has been removed.*

**Results with clinical samples:**

 For publications reporting the development of analytical methods, demonstration that the method works correctly on real clinical samples is highly desirable.

***Response:***

*We agree fully that a demonstration of the method performance with real clinical samples would be highly beneficial and a clinical study was also planned. However, due to several different circumstances, no clinical samples have been received as of this date.*

**Minor comments:**

- Abstract: ceftriaxone is a third generation cephalosporin (not antibiotic).
***Response:***
* Thank you for this suggestion. We have replaced, “antibiotic” with “cephalosporin”, in the abstract.*
- Internal standard is not "quantified.
***Response:***
* Thank you for this suggestion. We have replaced, “quantified” with “detected”.*
- Preparation of stock solutions: it should be specified whether the 10 mg/ml of stock solutions refer to the sodium salts or base drug.
***Response:***
* Thank you. Page 5, first paragraph: we have clarified that 10 mg/ml is the base form.*
- "Sample preparation" may be preferred to "sample extraction" as the preparation procedure consists in protein precipitation and phospholipid removal.
***Response:***
* Thank you for this suggestion. Page 5, third paragraph: we have changed “sample extraction” to “sample preparation”, as suggested. *

# Peer review: Reviewer response for version 2

**DOI:** 10.21956/wellcomeopenres.18966.r45734

**Published:** 2021-11-19

**Authors:** Sean O'Halloran

**Affiliations:** 1 School of Medicine, University of Western Australia, Perth, Australia

Thank you for revising the paper based on previous reviewer comments. I appreciate that some, but not all, of the previous comments have been addressed. 

 Previous comment regarding PKPD studies in neonates as an application but no case studies - not addressed.

 Previous comment about checking Tazobactam as an interference - the authors have not provided a satisfactory response. The authors' expectation that it is the local laboratory's responsibility to check interferences that might be present in a local population group or that just because Tazobactam in not co-administered in their country is an insufficient or unsatisfactory response for an international peer-review publication platform.

 Previous comment about recovery data being limited to Ceftriaxone - thank you for the authors' provision of data. The data is problematic. There is clearly a difference in recovery achieved for each compound which, again (corresponding to a previous comment) that the chosen internal standard is not satisfactory for this method. 

 Previous comment about lack of data on evaluating the isotopically-labelled version of Ceftriaxone - thank you for the authors' provision of data. The data is problematic. It is inappropriate practice to use a concentration of internal standard at an equivalent concentration to the LLOQ (1ug/mL). It is more appropriate to use an internal standard concentration, perhaps, midway, over the calibration range (in this case up to 155ug/mL).

 Previous comment about inability of the chosen internal standard to compensate for matrix effects - thank you for the authors' provision of a literature reference to Decosterd *et al.* Clearly the referenced method using an isotopically labelled internal standard demonstrates superior performance for this crucial parameter in LCMSMS method validation which suggests that the offered method is less worthy of publication. 

 Previous comment about the differing methodologies for evaluating matrix effects (post-column infusion versus post extract addition etc) - thank you for the authors' inclusion of Matuszewski's methodology. However results from utilisation of both methodologies are inconsistent. This suggests that the post-column infusion adds contradictory data to the validation of the method. 

 Previous comment regarding spelling errors - thank you to the authors for correcting many, but some persist. Example "were its third isotope...."

Is the rationale for developing the new method (or application) clearly explained?

Partly

Is the description of the method technically sound?

Partly

Are the conclusions about the method and its performance adequately supported by the findings presented in the article?

Partly

If any results are presented, are all the source data underlying the results available to ensure full reproducibility?

Partly

Are sufficient details provided to allow replication of the method development and its use by others?

Partly

Reviewer Expertise:

Chromatography, mass spectrometry, therapeutic drug monitoring, toxicology. pharmacology, drugs of abuse

I confirm that I have read this submission and believe that I have an appropriate level of expertise to state that I do not consider it to be of an acceptable scientific standard, for reasons outlined above.

# Peer review: Reviewer response for version 1

**DOI:** 10.21956/wellcomeopenres.16520.r35889

**Published:** 2019-07-19

**Authors:** Lotte van Andel

**Affiliations:** 1 Department of Pharmacy and Pharmacology, Antoni van Leeuwenhoek Hospital, Division of Pharmacology, Netherlands Cancer Institute, Amsterdam, The Netherlands

The authors have described a method to quantify ceftriaxone using HPLC-MS. The introduction gives a strong argument on the importance of a more sensitive method to quantify ceftriaxone. However, this argument loses its strength throughout the article. HPLC-MS is generally more sensitive than HPLC-UV, but the expected concentrations are quite high.  Moreover, an LLOQ of 1 µg/mL is not very low, hence I’d say that the method is not extremely sensitive. It would be interesting to know the LOD of the method.

 I wonder if the need for a sensitive method was related to the last concentrations measured in order to enable the construction of a pharmacokinetic profile? If these concentrations were likely to fall below the LLOQ of a HPLC-UV method, the estimation of the terminal phase might not be possible. Hence, the need for a more sensitive method. From the presented data, the concentration after 24 h is 5.3 – 15.1 μg/mL. It seems likely that these concentrations could be measured using an HPLC-UV method. Moreover, it is stated that some samples might be above ULOQ and need dilution before analysis. Furthermore, samples are diluted ~8 times before analysis. If a similar extraction procedure was tested by drying the samples and reconstituting them again in 100 µL, perhaps the samples could be measured on an HPLC-UV system instead.

 Finally, the authors claim that a disadvantage of HPLC-UV is the long run time of 10 – 20 min, whereas the current run time is 10 min. To me, this does not sound as a huge advantage over the other methods. The compound elutes at 1.6 min. Is the long run time required to stabilize the column? Has a shorter time been tested? Why are three mobile phases necessary? Elution occurs using mobile A only.

 It is unclear to me what the authors have learned from previously published methods. Also, a recent publication from 2018 has not been cited (Mohamed, 2018 ^1^). It would be valuable if the authors could clarify how the current method has been improved in comparison to the previously published methods. This could be added to the introduction to clarify the huge advantage of the current method over the previously published ones. The conclusion merely states that “The use of LC-MS/MS resulted in higher sensitivity and selectivity than HPLC-UV.” This could be explained further in the introduction or conclusion.

 Has it been investigated if the lipids interfered in LC-MS analysis? Otherwise phospholipid removal might not have been necessary, saving time and cost. Would phospholipid removal be necessary for HPLC-UV analysis? It would be valuable to know the extraction yield without the use of the phospholipid removal plates. Moreover, this step in the sample pretreatment could induce problems, because the internal standard used is not a stable isotopically labelled one. Based on accuracy and precision results it seems to correct for variation sufficiently. But I’d be careful to introduce more steps during sample preparation when a structural analogue is used rather than a stable isotopically standard.

 The authors have performed extensive interference analyses. However, to me it is not clear whether the interference between the analyte and IS was tested. This would have been useful. Moreover, the retention time of the internal standard is not specified.

 During sample processing, the samples are diluted ~8 times. If a similar extraction procedure was tested by drying the samples and reconstituting them again in 100 µL, could the samples be measured on an HPLC-UV system?

 The authors state “..revealing no significant suppression or enhancement”, after which they proceed to explain that QC1 exhibited a 27% signal enhancement. To me, this sounds contradictory. I’d suggest to remove the statement.

 The conclusion states that there might still be a limitation for infants if smaller amounts of blood are obtained. Therefore, it would be interesting to know the LOD of the method to assess whether the range could be widened.

 Some remarks:

- Acceptance criteria are not specifically stated.
- I wonder why there was a specific interest in testing different coagulants.
- It would be useful to know why the authors have methanol-washed pipette tips, well plates and seal mats before use.
- Some rephrasing needs to be done such as “To the first well which is the double blank was 400 µL..”.

Is the rationale for developing the new method (or application) clearly explained?

Partly

Is the description of the method technically sound?

Yes

Are the conclusions about the method and its performance adequately supported by the findings presented in the article?

Partly

If any results are presented, are all the source data underlying the results available to ensure full reproducibility?

Partly

Are sufficient details provided to allow replication of the method development and its use by others?

Yes

Reviewer Expertise:

LC-MS/MS, pharmacokinetics, mass balance, bioanalysis.

I confirm that I have read this submission and believe that I have an appropriate level of expertise to confirm that it is of an acceptable scientific standard, however I have significant reservations, as outlined above.

## References

[1] : Enhanced HPLC-MS/MS method for the quantitative determination of the co-administered drugs ceftriaxone sodium and lidocaine hydrochloride in human plasma following an intramuscular injection and application to a pharmacokinetic study. *Biomed Chromatogr* .2018;32(10) : 10.1002/bmc.4322 e4322 10.1002/bmc.4322 29934999

# Response

**Published:** 2021-08-20

**Authors:** Daniel Blessborn

**Affiliations:** Mahidol-Oxford Tropical Medicine Research Unit, Thailand

1. The authors have described a method to quantify ceftriaxone using HPLC-MS. The introduction gives a strong argument on the importance of a more sensitive method to quantify ceftriaxone. However, this argument loses its strength throughout the article. HPLC-MS is generally more sensitive than HPLC-UV, but the expected concentrations are quite high.  Moreover, an LLOQ of 1 µg/mL is not very low, hence I’d say that the method is not extremely sensitive. It would be interesting to know the LOD of the method.
**Response:**
** Thank you for this insightful response. We did not evaluate LOD as it is not a validation parameter.**
** As seen in figure 3, there was a small carryover effect. However, we verified that this carryover would not accumulate over time after repeated injections. Carryover was therefore tested in all 4 precision and accuracy batches, and was positioned to run after approximately 50 sample injections. As part of the validation, we tested an additional two concentration sets below the LLOQ; 0.5 µg/ml and 0.25 µg/ml. The results showed that 0.5 µg/ml would easily pass the carryover criteria as LLOQ. However, the 0.25 µg/ml would be too low to pass for LLOQ as the carryover was more than the 20% of the peak response. If there were no carryover, we would have pushed the sensitivity further. The chosen LOQ at 1.0 µg/ml produced robust and precise quantification, and should be sufficiently sensitive to evaluate the majority of pharmacokinetic studies.**
2. I wonder if the need for a sensitive method was related to the last concentrations measured in order to enable the construction of a pharmacokinetic profile? If these concentrations were likely to fall below the LLOQ of a HPLC-UV method, the estimation of the terminal phase might not be possible. Hence, the need for a more sensitive method. From the presented data, the concentration after 24 h is 5.3 – 15.1 μg/mL. It seems likely that these concentrations could be measured using an HPLC-UV method. Moreover, it is stated that some samples might be above ULOQ and need dilution before analysis. Furthermore, samples are diluted ~8 times before analysis. If a similar extraction procedure was tested by drying the samples and reconstituting them again in 100 µL, perhaps the samples could be measured on an HPLC-UV system instead.
**Response:**
** The concentrations (5.3 – 15.1 μg/ml) reported after 24 h is average values, which mean that 50% of all patients will have observed concentrations below and 50% above these reported values. Thus, you want to allow sufficient margin to enable quantification in the majority of patients that participate in the clinical trial.**
** Using LC-UV should be possible, and a publication released one year after ours, by Cairoli et al 2020 did just that. They could measure down to 1 µg/ml using 100µl plasma and LC-DAD with evaporation and reconstitution to 100 µl. Though matrix components would also be concentrated and could possibly pose an increased risk of interference.**
**  **
3. Finally, the authors claim that a disadvantage of HPLC-UV is the long run time of 10 – 20 min, whereas the current run time is 10 min. To me, this does not sound as a huge advantage over the other methods. The compound elutes at 1.6 min. Is the long run time required to stabilize the column? Has a shorter time been tested? Why are three mobile phases necessary? Elution occurs using mobile A only.
**Response:**
** The reviewer is correct, the elution occurs in isocratic mode using mobile phase A. Mobile phase B is used to flush out strongly retained components to avoid accumulation on the column, then Mobile phase C was added to help remove carryover and shorten the run time. Initially we had a 5-minute run time that included a washout gradient (A/B) to prevent more strongly retained components to accumulate on the column. However, there were still a carryover problem that was slowly reduced as the mobile phase flowed through the system and was eventually eliminated given enough time between injections (about 20 min). To reduce the waiting time between injections different washout solvents and solution mixes were tested, and by adding a washout step using ammonium bicarbonate, a final run-time of 10 minutes was achieved and the carry-over could be minimized but not completely eliminated.**
4. It is unclear to me what the authors have learned from previously published methods. Also, a recent publication from 2018 has not been cited (Mohamed, 2018 ^1^ ). It would be valuable if the authors could clarify how the current method has been improved in comparison to the previously published methods. This could be added to the introduction to clarify the huge advantage of the current method over the previously published ones. The conclusion merely states that “The use of LC-MS/MS resulted in higher sensitivity and selectivity than HPLC-UV.” This could be explained further in the introduction or conclusion.
**Response:**
** The publication by Mohammed 2018 have now been cited in the introduction, and their method uses 450µl of sample and achieved a LOQ of 3 µg/ml. However, we do not claim that we have a superior method but merely an alternative option to already published methods. Also, many published ceftriaxone methods describe matrix effects, but not all of them have quantified these effects. Furthermore, methods report carryover result and many methods have short retention time but very long run time without explaining the reason. Some methods with short run times have included repeat injections of blank samples to reduce carryover, which makes the method unsuitable for routine analysis of clinical studies. We wanted to be clear and describe the problems we have encountered, as well as the different strengths and weaknesses and we hope that this information can help others setting up a method to quantify ceftriaxone**.
**Also, after our publication, there were three new publications in 2020, two LC-MS and one DAD-UV using the same sample size (100µl) and similar calibration range. Both LC-MS methods experienced matrix effects, and one of them (Decosterd et.al. 2020) used ceftriaxone-13CD _3_ that showed that it could compensate for the 150-175% matrix effects they otherwise would have experienced, and we have added this in the discussion section. Though they did not show any evaluation of interference between analyte and SIL-IS, or if the isotopes of ceftriaxone ads to the ceftriaxone-13CD _3_ signal as it did in our evaluation of ceftriaxone-D _3_. Theoretical predictions show that some interference could be present in small amounts, but their ULOQ go only to 100 µg/ml which would limit these effects. **
5. Has it been investigated if the lipids interfered in LC-MS analysis? Otherwise phospholipid removal might not have been necessary, saving time and cost. Would phospholipid removal be necessary for HPLC-UV analysis? It would be valuable to know the extraction yield without the use of the phospholipid removal plates. Moreover, this step in the sample pretreatment could induce problems, because the internal standard used is not a stable isotopically labelled one. Based on accuracy and precision results it seems to correct for variation sufficiently. But I’d be careful to introduce more steps during sample preparation when a structural analogue is used rather than a stable isotopically standard. 
**Response: Instrument and chromatographic conditions in the result section and conclusion section has been edited to clarify the reasons for phospholipid removal (also see the response of last question from reviewer 1). Briefly, phospholipid removal generally reduces matrix components due to removal of phospholipids and large particles. In early method development, we compared protein precipitation with different types of extraction plates. By using this type of sample cleanup, you can also reduce the risk of particles clogging the system causing analytical failures during the analytical run, and the risk of accumulation of strongly retaining components on the column causing deterioration of column performance. In our experience this will maintain the column performance and reduce the risk of analytical problems during the analysis and the need of re-analysis.**
6. The authors have performed extensive interference analyses. However, to me it is not clear whether the interference between the analyte and IS was tested. This would have been useful. Moreover, the retention time of the internal standard is not specified.
**Response:**
** More information on the interference testing between analyte and SIL-D _3_ have been added to the manuscript. Retention time of the new internal standard is now shown in the updated figure 3.**
7. During sample processing, the samples are diluted ~8 times. If a similar extraction procedure was tested by drying the samples and reconstituting them again in 100 µL, could the samples be measured on an HPLC-UV system?
**Response:**
** Also see our response on comment 2 above. LC-UV should be possible, as seen in a publication released one year after ours (Cairoli et al 2020). They measured down to 1 µg/ml (LOQ) using 100 µl plasma and LC-DAD with evaporation and reconstitution to 100 µl. However, matrix components would also be concentrated and could possibly pose an increased risk of interference.**
8. The authors state “..revealing no significant suppression or enhancement”, after which they proceed to explain that QC1 exhibited a 27% signal enhancement. To me, this sounds contradictory. I’d suggest to remove the statement.
**Response:**
** This statement have been removed and the matrix effect section have been re-written to clarify the validation findings.**
9. The conclusion states that there might still be a limitation for infants if smaller amounts of blood are obtained. Therefore, it would be interesting to know the LOD of the method to assess whether the range could be widened.
**Response:**
** Also see our response on comment 1 above. We did not evaluate LOD as it is not a validation parameter. **
**The results showed that 0.5 µg/ml would easily pass the carryover criteria as LLOQ. However, the 0.25 µg/ml would be too low to pass for LLOQ as the carryover was more than the 20% of the peak response. The chosen LOQ at 1.0 µg/ml produced robust and precise quantification, and should be sufficiently sensitive to evaluate the majority of pharmacokinetic studies. The range could possibly be extended to 0.5 µg/ml, but this would require a re-validation.**

Some remarks:

- Acceptance criteria are not specifically stated.
**Response:**
** Acceptance criteria has been added to method section. **
- I wonder why there was a specific interest in testing different coagulants.
**Response:**
** From our experience we know that some studies want to streamline and make sample collection easy by using one anticoagulant for different measurements. The most commonly used anticoagulants are heparin and EDTA, so we test different anticoagulants in the validation to see if they are comparable and if a certain anticoagulant can be used. FDA guidelines also require validating the anticoagulants to be used. **
- It would be useful to know why the authors have methanol-washed pipette tips, well plates and seal mats before use.
**Response:**
** The methanol washed tips and labware was part of the matrix effects investigation but it did not have a noticeable effect so the sentence has been removed in the updated manuscript.**
  Some rephrasing needs to be done such as “To the first well which is the double blank was 400 µL..”.
**Response:**
**The sentence has been re-written.**

# Peer review: Reviewer response for version 1

**DOI:** 10.21956/wellcomeopenres.16520.r35674

**Published:** 2019-07-04

**Authors:** Sean O'Halloran

**Affiliations:** 1 School of Medicine, University of Western Australia, Perth, Australia

This manuscript outlines the determination of Ceftriaxone in human plasma by LCMSMS. The authors mention that a potential application of this method relates to performance of PKPD studies in neonates and malnourished young children however they do not include any case studies. The authors’ stated objective is to offer this method to laboratories setting up this LCMSMS assay. Such literature can be important and useful but in this case the authors need to be very clear in their abstract about the particular details of the method, including any limitations, so that prospective laboratories can indeed assess the suitability of this method for their local application. As such, the abstract must include the finding that calibrators must match the anticoagulant used for specimen collection. 

   

 The authors claim, repetitively, that their validation is in accordance with FDA guidelines. Method validation protocols generally discourage the use of serial dilution of calibration material or stock.  In the methodology description of selectivity by other compounds the author declare that the ‘occurrence of a peak response at the retention time of the analyte or internal standard indicates matrix interference’. This is incorrect; such interference could be from a number of sources, not just matrix. In the same description there is lack of specific detail of the concentration of infused solution and concentration of potentially-interfering substances in the post-column infusion study.  The authors must cite references that describe that this methodology is suitable for interference studies from specific compounds such as co-administered drugs. There are formulations of Ceftriaxone, albeit in specific countries only, that contain Tazobactam, so this is among other compounds that need to be specifically tested for interference.   

 The authors need to separate description and results of the matrix effects study from the carryover study into different sections. The presentation of the results of the matrix effects study is not clear. The authors use the phrase ‘post-spiked’ blank plasma however the literature tends to use the terminology ‘post-extract addition’. The authors describe the use of neat reference solution however they do not describe in detail how that solution was prepared. A presentation of the matrix effects study results should consider methodology by Matuszewski et al. ^1^. While their description of comparison between neat solution and post-extract spiked solution is correct for describing a matrix effect-study, the authors then go on to describe matrix effects in a QC sample. This needs clarification. The word ‘tendency’ is not useful. These descriptions of enhancement and suppression contradict the assertion in the abstract that no significant (poor choice of word) matrix effects were observed. The finding that Ceftriaxone is subject to matrix effects and the internal standard Cefotaxime is not, suggests that Cefotaxime is not suitable as an internal standard.     

 The standard and the internal standard were dissolved in two different solvents suggesting differing physicochemical properties; their relative and respective retention times in the method need to be made more explicit. In describing the evaluation of a labelled internal standard the authors do not make it clear whether or not they had purchased the labelled material, or that they had used the purchased labelled material (not mentioned in Materials and Reagents) to determine the MRM transitions by tuning for the D3 internal standard. Or did they rely only on the theoretical abundance of natural isotopes thereby dismissing the suitability of the compound? There is lack of information on the various product ions suitable for monitoring the D3 compound and whether or not the deuterium atoms are thought to be on those product ion fragments. CLSI tolerates 5% of the labelled internal standard signal at the ULOQ, and CLSI suggests that labelled compounds +3 mass units should suit analytes of molecular mass <1000. Also, under “Validation” the description of the recovery study is not clear, dealing with Ceftriaxone only. Evaluation of the internal standard recovery must also be included.

 The authors declare that ‘higher reproducibility was achieved with acetonitrile’ to ‘improve the sample purity’, this is incorrect, and furthermore, reproducibility as an analytical method validation parameter is not presented anywhere in the paper, either in methodology or results.

 The authors admit some kind of problem with carryover. Detail must be provided. Increasing the run time to 10 minutes is presented as a way of dealing with carryover; this parameter must be presented in the abstract because the run-time is consequently within the range of HPLC assay run times – methodology and technology that is supposed to be surpassed by this LCMSMS method. 

 The abstract must also include the requirement for 100 µL of sample which does not compare favourably with the requirement of HPLC methods and where other authors (Page-Sharp et al ^2^) require lower sampling volumes for paediatric patients.

 This version of the manuscript must be edited further.   

 Ethical approval was sought; is not clear that the approval was given, please address.

 Please clarify the use of a binary pump with three different mobile phases.

 There is some repetition in the manuscript which must be removed. 

 In many parts of the manuscript there is a mixture of methodology and results, which needs to be corrected.   

 In the “Conclusion” section, the authors suggest that ‘phospholipid removal techniques for sample preparation could reduce some matrix interferences’. This is unclear because the authors have already described such a phospholipid removal technique in the method they have presented.

 In the description of the extraction procedure the authors detail ‘To the first well which is the double blank was 400 µL of acetonitrile added’. This and other such phrases need to be edited and re-worded.  

 Other phrases or words that must be edited in their respective contexts include words like ‘mainly’, ‘significant’, ‘a few’ ,‘only through’, ‘several’, ‘among’, ‘tendency’ ‘some degree’, ‘in the pipeline’ etc. Typo example, ‘were some isotopes’.

Is the rationale for developing the new method (or application) clearly explained?

Partly

Is the description of the method technically sound?

Partly

Are the conclusions about the method and its performance adequately supported by the findings presented in the article?

Partly

If any results are presented, are all the source data underlying the results available to ensure full reproducibility?

Partly

Are sufficient details provided to allow replication of the method development and its use by others?

Partly

Reviewer Expertise:

Chromatography, mass spectrometry, therapeutic drug monitoring, toxicology. pharmacology, drugs of abuse

I confirm that I have read this submission and believe that I have an appropriate level of expertise to confirm that it is of an acceptable scientific standard, however I have significant reservations, as outlined above.

## References

[1] : Strategies for the Assessment of Matrix Effect in Quantitative Bioanalytical Methods Based on HPLC−MS/MS. *Analytical Chemistry* .2003;75(13) : 10.1021/ac020361s 3019-3030 10.1021/ac020361s 12964746

[2] Liquid Chromatography - Mass Spectrometry Methods;Approved Guideline. *Clinical Laboratory standards Institute* .2014;34(16) : Reference source

# Response

**Published:** 2021-08-20

**Authors:** Daniel Blessborn

**Affiliations:** Mahidol-Oxford Tropical Medicine Research Unit, Thailand

1. This manuscript outlines the determination of Ceftriaxone in human plasma by LCMSMS. The authors mention that a potential application of this method relates to performance of PKPD studies in neonates and malnourished young children however they do not include any case studies. The authors’ stated objective is to offer this method to laboratories setting up this LCMSMS assay. Such literature can be important and useful but in this case the authors need to be very clear in their abstract about the particular details of the method, including any limitations, so that prospective laboratories can indeed assess the suitability of this method for their local application. As such, the abstract must include the finding that calibrators must match the anticoagulant used for specimen collection. 
**Response:**
** Thank you for your suggestions. We have added additional information to the abstract, such as anticoagulant must match calibrators and collected specimen and that CPD plasma resulted in matrix enhancement in the low concentration level.   **
    
2. The authors claim, repetitively, that their validation is in accordance with FDA guidelines. Method validation protocols generally discourage the use of serial dilution of calibration material or stock.  In the methodology description of selectivity by other compounds the author declare that the ‘occurrence of a peak response at the retention time of the analyte or internal standard indicates matrix interference’. This is incorrect; such interference could be from a number of sources, not just matrix. In the same description there is lack of specific detail of the concentration of infused solution and concentration of potentially-interfering substances in the post-column infusion study.  The authors must cite references that describe that this methodology is suitable for interference studies from specific compounds such as co-administered drugs. There are formulations of Ceftriaxone, albeit in specific countries only, that contain Tazobactam, so this is among other compounds that need to be specifically tested for interference.   
**Response:**
** Thank you for these insightful comments. To the best of our knowledge, regulatory guidelines (e.g. EMA 2012 and FDA 2018) does not mention exactly how to prepare different calibrators (i.e. serial or direct dilutions), but they do specify that QC samples should be prepared from a separate stock solution, independently from calibration standards. We followed these guidelines, and used two independently prepared stock solutions for calibrators and QC samples. We believe that this is an acceptable approach.**
**The sentence ‘occurrence of a peak response at the retention time of the analyte or internal standard indicates matrix interference’ have been corrected and now state that “ *The occurrence of a peak response at the retention time of the analyte or internal standard indicates an interference and would require further investigation.*”**
** We have also added the concentration of infused ceftriaxone and internal standard, and the concentration of potentially interfering drugs injected during post-column infusion (as suggested by reviewer). Tazobactam was not considered as it is not co-formulated in marketed products in the country we are located (Thailand). There are also other drug combinations, used in specific countries, and these needs to be evaluated by the analytical laboratory setting up the method depending on the specific requirements for the samples that will be analysed. We believe that it is beyond the scope of this paper to evaluate all possible combinations that might be of interest for different laboratories. **
3. The authors need to separate description and results of the matrix effects study from the carryover study into different sections. The presentation of the results of the matrix effects study is not clear. The authors use the phrase ‘post-spiked’ blank plasma however the literature tends to use the terminology ‘post-extract addition’. The authors describe the use of neat reference solution however they do not describe in detail how that solution was prepared. A presentation of the matrix effects study results should consider methodology by Matuszewski et al. ^1^ . While their description of comparison between neat solution and post-extract spiked solution is correct for describing a matrix effect-study, the authors then go on to describe matrix effects in a QC sample. This needs clarification. The word ‘tendency’ is not useful. These descriptions of enhancement and suppression contradict the assertion in the abstract that no significant (poor choice of word) matrix effects were observed. The finding that Ceftriaxone is subject to matrix effects and the internal standard Cefotaxime is not, suggests that Cefotaxime is not suitable as an internal standard.
**Response: Thank you for your comments and suggestions to improve the manuscript. We have now rewritten that particular section, and separated carry-over effects from the text on matrix effects. The matrix effect investigation is in fact based on the article of Matuszewski et al. (2003), but follows the simplified approach presented in the same article. This approach uses two concentration levels of the analyte in 6 different sources of blank plasma. We have rephrased the terminology as suggested, and clarified the matrix effect evaluation was in accordance to Matuszewski et al. The sentence describing a matrix effect in a QC sample was indeed a mistake and we are grateful that the reviewer pointed out this typing error (this has been corrected). Additional words and sentences have also been changed as suggested by the reviewer, and we hope that these edits now provide a clear and accurate method description. **
4. The standard and the internal standard were dissolved in two different solvents suggesting differing physicochemical properties; their relative and respective retention times in the method need to be made more explicit. In describing the evaluation of a labelled internal standard the authors do not make it clear whether or not they had purchased the labelled material, or that they had used the purchased labelled material (not mentioned in Materials and Reagents) to determine the MRM transitions by tuning for the D3 internal standard. Or did they rely only on the theoretical abundance of natural isotopes thereby dismissing the suitability of the compound? There is lack of information on the various product ions suitable for monitoring the D3 compound and whether or not the deuterium atoms are thought to be on those product ion fragments. CLSI tolerates 5% of the labelled internal standard signal at the ULOQ, and CLSI suggests that labelled compounds +3 mass units should suit analytes of molecular mass <1000. Also, under “Validation” the description of the recovery study is not clear, dealing with Ceftriaxone only. Evaluation of the internal standard recovery must also be included.
**Response: The theoretical physicochemical properties of ceftriaxone (standard) and cefotaxime (internal standard) are similar, and both have very similar theoretical logD curves over the pH range. Both compounds can be dissolved in water, and the retention and separation are relatively close when using an isocratic mode (as can be seen in the revised figure 3). However, we decided to follow the CoA provided from the supplier when dissolving the standard and internal standard, thus explain why we used different solvents. Regarding the labelled internal standard (ceftriaxone-D _3_), the source of this compound has been added in the material section, and we have restructured and re-written “Result and discussion – Instrument and chromatographic condition section” to explain better why ceftriaxone-D _3_ was excluded (i.e. the level of interference between ceftriaxone and ceftriaxone-D _3_). A SIL-IS of D _5_ or higher would most likely work, but we have not been able to find such a labelled internal standard. Recovery of the new alternative internal standard (cefotaxime) have been added in the text as suggested, and the retention time and separation can now be seen in the updated figure 3.**
5. The authors declare that ‘higher reproducibility was achieved with acetonitrile’ to ‘improve the sample purity’, this is incorrect, and furthermore, reproducibility as an analytical method validation parameter is not presented anywhere in the paper, either in methodology or results.
  The authors admit some kind of problem with carryover. Detail must be provided. Increasing the run time to 10 minutes is presented as a way of dealing with carryover; this parameter must be presented in the abstract because the run-time is consequently within the range of HPLC assay run times – methodology and technology that is supposed to be surpassed by this LCMSMS method. 
  The abstract must also include the requirement for 100 µL of sample which does not compare favourably with the requirement of HPLC methods and where other authors (Page-Sharp et al ^2^ ) require lower sampling volumes for paediatric patients.
**Response:**
** We fully agree that this sentence was unclear, and it has been edited for clarity in the revised manuscript. Details of the carry-over problem and how it was solved, although resulting in a longer run time, has now been added in the discussion. The analytical run time and sample volume required has been added in the abstract, as suggested.**
6. This version of the manuscript must be edited further. Ethical approval was sought; is not clear that the approval was given, please address. Please clarify the use of a binary pump with three different mobile phases. There is some repetition in the manuscript which must be removed.  In many parts of the manuscript there is a mixture of methodology and results, which needs to be corrected.   
**Response:**
** We thank the reviewer for highlighting these errors in the manuscript. Indeed, ethical approval was given and we have clarified this in the revised manuscript. The LC-pump was a quaternary pump (not a binary pump) and this has been corrected in the text. We have also re-arranged the text in the method and result sections as suggested by the reviewer.**
7. In the “Conclusion” section, the authors suggest that ‘phospholipid removal techniques for sample preparation could reduce some matrix interferences’. This is unclear because the authors have already described such a phospholipid removal technique in the method they have presented.
  In the description of the extraction procedure the authors detail ‘To the first well which is the double blank was 400 µL of acetonitrile added’. This and other such phrases need to be edited and re-worded.  
  Other phrases or words that must be edited in their respective contexts include words like ‘mainly’, ‘significant’, ‘a few’ ,‘only through’, ‘several’, ‘among’, ‘tendency’ ‘some degree’, ‘in the pipeline’ etc. Typo example, ‘were some isotopes’.
**Response:**
** The conclusion section has been edited and clarified to state that phospholipid plates commonly reduce matrix components due to removal of phospholipids, but also other particles. By using this type of sample cleanup, you can reduce the risk of particles clogging the system causing analytical failures during the run as well as reducing the amount of matrix components passing through the column. The description of the extraction procedure was also rewritten as suggested. Other phrases and words listed by the reviewer have been edited.**
